# Supplementary material for: Comparative efficacy and acceptability of different antihypertensive drug classes for cardiovascular disease prevention: protocol for a systematic review and network meta-analysis
Source: BMJ Open. 2021 Mar 27;11(3):e044302. doi: 10.1136/bmjopen-2020-044302 (PMC8009235; doi:10.1136/bmjopen-2020-044302)
Supplement: Author's manuscript [file bmjopen-2020-044302.draft_revisions.pdf]

BMJ Open

BMJ Open is committed to open peer review. As part of this commitment we make the peer review history of every article we publish publicly available.

When an article is published we post the peer reviewers' comments and the authors' responses online. We also post the versions of the paper that were used during peer review. These are the versions that the peer review comments apply to.

The versions of the paper that follow are the versions that were submitted during the peer review process. They are not the versions of record or the final published versions. They should not be cited or distributed as the published version of this manuscript.

BMJ Open is an open access journal and the full, final, typeset and author-corrected version of record of the manuscript is available on our site with no access controls, subscription charges or pay-per-view fees (http://bmjopen.bmj.com).

If you have any questions on BMJ Open's open peer review process please email info.bmjopen@bmj.com

BMJ Open

Comparative efficacy and acceptability of different antihypertensive drug classes for cardiovascular disease prevention – protocol for a systematic review and network meta-analysis

Journal:	*BMJ Open*
Manuscript ID	bmjopen-2020-044302
Article Type:	Protocol
Date Submitted by the Author:	01-Sep-2020

Complete List of Authors:	Jussil, Heidi; Umeå Universitet Medicinska fakulteten, Department of Public Health and Clinical Medicine<br>Chaimani, Anna; Universite de Paris, Research Center of Epidemiology (CRESS-UMR1153), INSERM, INRA<br>Carlberg, Bo; Umeå Universitet Medicinska fakulteten, Department of Public Health and Clinical Medicine<br>Brunström, Mattias ; Umeå Universitet Medicinska fakulteten, Department of Public Health and Clinical Medicine

Keywords:	Hypertension < CARDIOLOGY, Cardiology < INTERNAL MEDICINE, Stroke medicine < INTERNAL MEDICINE, Vascular medicine < INTERNAL MEDICINE

SCHOLARONE™ Manuscripts

I, the Submitting Author has the right to grant and does grant on behalf of all authors of the Work (as defined in the below author licence), an exclusive licence and/or a non-exclusive licence for contributions from authors who are: i) UK Crown employees; ii) where BMJ has agreed a CC-BY licence shall apply, and/or iii) in accordance with the terms applicable for US Federal Government officers or employees acting as part of their official duties; on a worldwide, perpetual, irrevocable, royalty-free basis to BMJ Publishing Group Ltd ("BMJ") its licensees and where the relevant Journal is co-owned by BMJ to the co-owners of the Journal, to publish the Work in this journal and any other BMJ products and to exploit all rights, as set out in our licence.

The Submitting Author accepts and understands that any supply made under these terms is made by BMJ to the Submitting Author unless you are acting as an employee on behalf of your employer or a postgraduate student of an affiliated institution which is paying any applicable article publishing charge ("APC") for Open Access articles. Where the Submitting Author wishes to make the Work available on an Open Access basis (and intends to pay the relevant APC), the terms of reuse of such Open Access shall be governed by a Creative Commons licence – details of these licences and which Creative Commons licence will apply to this Work are set out in our licence referred to above.

Other than as permitted in any relevant BMJ Author's Self Archiving Policies, I confirm this Work has not been accepted for publication elsewhere, is not being considered for publication elsewhere and does not duplicate material already published. I confirm all authors consent to publication of this Work and authorise the granting of this licence.

For peer review only

Comparative efficacy and acceptability of different antihypertensive drug classes for cardiovascular disease prevention – protocol for a systematic review and network meta-analysis

Heidi Jussil[1], Anna Chaimani[2], Bo Carlberg[1], Mattias Brunström[1]

[1] Department of Public Health and Clinical Medicine, Umeå University, Umeå, Sweden
[2] Université de Paris, Research Center of Epidemiology and Statistics (CRESS-U1153), INSERM, INRA, F-75004 Paris, France

Correspondence:
Dr Mattias Brunström
Dept. Public Health and Clinical Medicine
Umeå University
901 87, Umeå
Sweden
mattias.brunstrom@umu.se
+46733693182

3 279 words
1 table
1 figure

For peer review only

Abstract

Introduction

Clinical practice guidelines differ in their recommendations on first-line antihypertensive drug classes. No adequately powered randomized controlled trial have assessed all major drug classes against each other, and previous meta-analyses have mainly relied on pairwise meta-analyses for treatment comparisons.

Methods and analysis

A systematic review and network meta-analysis will be carried out to assess the efficacy and acceptability of all major antihypertensive drug classes. PubMed and CENTRAL were searched on Februray 21st 2020 to identify randomized controlled trials with at least 1000 person-years of follow-up, assessing any antihypertensive agent against other agents or placebo. All trials fulfilling the inclusion criteria will be assessed for risk of bias using the second version of Cochrane's risk of bias assessment tool. The study selection process, risk of bias assessment, and data extraction is done by two authors in duplicate. Relative risks from individual trials will be combined in pairwise meta-analyses; in the absence of important intransitivity, random-effects network meta-analysis will be performed. The primary outcome for efficacy will be major adverse cardiovascular events, whereas the primary acceptability outcome will be treatment discontinuation for any reason. Additional outcomes include all-cause mortality, cardiovascular mortality, stroke, myocardial infarction, heart failure, and acute renal failure. The impact of differences within drug classes will be

For peer review only

explored through alternative networks, including analyzing thiazide-like and thiazide-type diuretics separately.

Ethics and dissemination

This review will only process aggregated study level data and does not require ethical approval. The findings will be published in a peer-reviewed medical journal.

Registration

The protocol was registered in PROSPERO August 21st 2020. *Registration number will be added once received.*

For peer review only

Strengths and limitations of this study

- This systematic review and network meta-analysis will be the first contemporary review to assess the efficacy and acceptability of all major antihypertensive drug classes, incorporating direct and indirect evidence
- Broad inclusion criteria in terms of patient characteristics ensure wide applicability of the study findings; potential differences between patient groups will be explored through subgroup- and sensitivity analyses.
- Alternative networks, with different node definitions, will be used to explore differences within drug classes, specifically differences between thiazide-like and thiazide-type diuretics.
- The main limitation of this review is the use of study-level data, with no possibility to account for individual patient characteristics in the analyses.

For peer review only

Introduction

Hypertension (blood pressure ≥ 140/90 mmHg) is estimated to affect more than one billion people globally.[1,2] Studies have shown that hypertension is the most important risk factor for death and cardiovascular disease (CVD) worldwide[2], and that lowering blood pressure in the hypertensive range reduces the risk of CVD.[2,3]

Five major drug classes have been shown to reduce the risk of CVD compared to placebo or no treatment; angiotensin-converting enzyme inhibitors (ACEi), angiotensin II receptor blockers (ARB), calcium channel blockers (CCB), diuretics and beta blockers (BB).[4,5] Although beta-blockers have generally been abandoned as first-line therapy due to less effective stroke prevention compared to other agents[6], current hypertension guidelines differ in the recommendations on what drug class to use as first-line therapy.[7-9] Whereas US guidelines recommend single therapy with ACEi, ARB, CCB or diuretics initially[7], European guidelines recommended to start treatment with a renin-angiotensin aldosterone system (RAAS) inhibitor (ACEi or ARB), in combination with a CCB or a diuretic.[8] The most recent UK recommendations, from the National Institute for Health and Care Excellence (NICE), support RAAS inhibitors in young people and people with diabetes, whereas CCB:s are considered the drug class of choice for elderly and people of African heritage.[9]

Even though many antihypertensive agents have been compared to one another or placebo separately, no adequately powered trial has compared all major drug classes head-to-head, assessing CVD outcomes. Several systematic reviews and pairwise meta-analyses aiming to assess this have been published, but all have limited their analyses to comparative trials

For peer review only - http://bmjopen.bmj.com/site/about/guidelines.xhtml

assessing two or more agents against each other, with the exclusion of placebo-controlled trials.[5,10-12] Excluding placebo-controlled trials means exclusion of potentially valuable information gained through indirect comparisons, likely affecting the overall results.[13] Further, the two most cited meta-analyses supporting the view that all major drug classes are comparable have used pairwise meta-analyses, comparing each drug class to all other drug classes.[5,10] This implicitly assumes that all drug classes used for comparison are similar, which may conceal clinically important differences. The only contemporary network meta-analysis assessing different antihypertensive agents in the general population to date, is hard to interpret due to limitations in study selection, data extraction and risk of bias assessment.[14]

All major systematic reviews have used the conventional classification of antihypertensive agents described above.[4,5,10-12] However, categorization of drug classes might play a critical role for the results of network meta-analyses. For example, the class of diuretics may be split into thiazide-type and thiazide-like diuretics. US and European guidelines equate these subclasses[7,8], whereas the UK guidelines preferentially recommend thiazide-like diuretics.[9] The latter recommendation is supported by pharmacological differences[15], as well as recent meta-analyses assessing differences in blood pressure-lowering and CVD reduction[16,17]. Another categorization of interest is that of RAAS inhibitors (ACEi and ARB). Although pharmacologically distinct, these classes act on the same physiological system, and evidence from clinical trials suggests no difference in efficacy between classes.[18] Thirdly, CCB can be subdivided into dihydropyridine and non-dihydropyridine agents, the former being highly selective for the vasculature whereas the latter also has antiarrhythmic properties.[19]

For peer review only

In summary, clinical practice guidelines differ in their recommendations on first-line antihypertensive treatment. This reflects the absence of head-to-head comparisons between major drug classes, and hence uncertainty regarding the comparative efficacy between classes. The aim and purpose of this study is to compare the efficacy and acceptability of different antihypertensive drug classes for CVD prevention through a systematic review and network meta-analysis, incorporating evidence from comparative as well as placebo-controlled trials. Further, we will explore the potential differences among different subclasses of diuretics, RAAS-inhibitors, and CCB.

Methods

We will perform a systematic review guided by the recommendations from the Cochrane Collaboration.[20] The protocol is written with guidance from the PRISMA-P statement, with additional considerations specific for network meta-analyses[21,22], and was registered in the PROSPERO database 21st of August 2020 *(CDR number will be added when received – delay due to Covid)*. Any important updates to the protocol will be amended to PROSPERO, and/or described in the final report. The final report will follow the recommendations from the PRISMA-NMA statement.[23]

Eligibility criteria

Types of studies

Randomized controlled trials comparing any single antihypertensive agent, or combination of antihypertensive agents, against another agent, combination of agents, placebo, or no

For peer review only - http://bmjopen.bmj.com/site/about/guidelines.xhtml

treatment will be included. Trials using a prospective randomized open-label blinded endpoint (PROBE) design will be accepted, whereas cluster-randomized trials, crossover trials, and trials of combined or complex interventions will be excluded. We will restrict our analyses to trials with at least 1000 person-years of follow-up to avoid small trials not primarily assessing the effect of blood pressure-lowering agents on cardiovascular events, but rather the effect of different agents on blood pressure levels. Hundreds of such trials exist; cardiovascular events are uncommon, and when present recorded as adverse events and therefore not properly adjudicated.

For peer review only

Types of participants

Trials will be included if participants are either primary preventive, have stable coronary artery disease, previous cerebrovascular disease, diabetes mellitus, or mild chronic kidney disease (eGFR 60-89 ml/min/1.73m^2). Because the main drug classes listed below (see "*types of intervention"* section) may be combined (except for ACEi + ARB; combination excluded), and prescribed in any order without important interactions on cardiovascular outcome level, we will include trials of treatment naïve patients and previously treated patients in the network meta-analysis. Studies including more than 50% of participants with heart failure, left ventricular dysfunction, acute myocardial infarction, macroalbuminuria, end-stage renal disease, or chronic kidney disease stage 3 or higher (eGFR < 60 ml/min/1.73m^2) at baseline will be excluded. The reason for these exclusions is that one or several of the included drug classes have specific effects in these patient groups, that possibly goes beyond blood pressure lowering.[7,8,24,25] Thus, the transitivity assumption (see "*Evaluation of the transitivity assumption*" section below) would be violated if such trials were combined with trials

For peer review only - http://bmjopen.bmj.com/site/about/guidelines.xhtml

assessing similar agents in patient populations where such effects are absent. Although some would argue for specific effects of RAAS inhibitors in people with stable coronary artery disease, as well as in people with diabetes and mild chronic kidney disease, recent systematic reviews have not confirmed such an effect on cardiovascular outcomes.[18,26-28] Based on these findings, trials in people with stable coronary artery disease, diabetes and mild chronic kidney disease will be included in our main analysis, and excluded in sensitivity analyses to assess the impact on the overall results.

Types of intervention

The main interventions of interest are antihypertensive drug classes which have previously been shown to reduce the risk of cardiovascular events; ACE, ARB, BB, CCB, and diuretics. These are the main treatment options in clinical practice for the types of participants listed above (se "*types of participants*" section), and will therefore constitute the decision set of interventions in this review. Other drug classes, such as potassium-sparing diuretics, alpha-blockers, peripheral vasodilators, and central-acting agents, as well as drug combinations including any of the above-mentioned drug classes, placebo, and no treatment, will be included in the network as supplementary interventions to maximize the amount of evidence through indirect comparisons (Figure 1).

Outcome measures

The primary outcome for efficacy will be the composite outcome, major adverse cardiovascular events (MACE). Definitions of MACE vary to some extent across trials, but commonly include cardiovascular mortality, stroke, and myocardial infarction. Inclusion of

For peer review only - http://bmjopen.bmj.com/site/about/guidelines.xhtml

heart failure, revascularisation, and peripheral artery disease differ across trials. If available, we prefer the strict definition using cardiovascular mortality, myocardial infarction, and stroke. If other definitions of MACE have been applied, they will generally be accepted but appropriateness for inclusion will be assessed on a case-by-case basis. All deviations from the preferred definition will be denoted, and the impact of MACE definition will be explored in sensitivity analyses, excluding trials not presenting results according to the preferred definition.

The primary outcome for acceptability will be permanent discontinuation of study medication. We will include discontinuations for any reason because this reflects safety as well as efficacy, and because the reporting of reasons for discontinuation differs to a large extent between trials.

Secondary outcomes are all-cause mortality, cardiovascular mortality, myocardial infarction, stroke, heart failure, and acute kidney injury. Mortality outcomes, as well as myocardial infarction and stroke, have generally accepted universal definitions, although for myocardial infarction the definition has changed slightly over the years. For these outcomes, the definitions applied in individual trials will generally be accepted. For myocardial infarction and stroke we primarily aim to include fatal and non-fatal events, combined. If only fatal or non-fatal events are reported separately, however, we will accept and include the reported numbers. We will also accept “acute coronary syndrome” as equivalent to myocardial infarction, but we will not include composite coronary events including revascularizations. For heart failure, we will accept definitions including new diagnosis of heart failure, heart failure hospitalizations, and heart failure mortality. For acute kidney injury, we will apply the

For peer review only - http://bmjopen.bmj.com/site/about/guidelines.xhtml

Kidney Disease: Improving Global Outcomes (KDIGO) definition, including at least 50 % increase in serum creatinine, where appropriate. However, we will also include discontinuation of treatment due to kidney injury, as many trials were designed before the KDIGO definition of acute kidney injury was published.

Search strategy and study selection

Preliminary searches in PubMed and CENTRAL were performed on February 21st 2020. We used search terms for blood pressure lowering treatment combined with several cardiovascular disease terms in both databases. We restricted our search to randomized controlled trials, but applied no date or language restrictions.

Full search strategy in PubMed was (("antihypertensive agents"[Pharmacological Action] OR "antihypertensive agents"[MeSH Terms] OR "antihypertensive"[All Fields] OR "blood pressure-lowering"[All Fields] OR "blood pressure lowering"[All Fields] OR "blood-pressure lowering"[All Fields]) AND ("cardiovascular"[All Fields] OR "myocardial"[All Fields] OR "stroke"[MeSH Terms] OR "stroke"[All Fields] OR "heart failure"[All Fields] OR "mortality"[All Fields]) AND (randomized controlled trial[Publication Type] OR (randomized[Title/Abstract] AND controlled[Title/Abstract] AND trial[Title/Abstract])).

Full search strategy for CENTRAL was (antihypertensive near agents OR "blood pressure-lowering" OR "blood pressure lowering" OR "blood-pressure lowering") AND ("cardiovascular" OR "myocardial" OR "stroke" OR "heart failure" OR "mortality") in All Text.

For peer review only - http://bmjopen.bmj.com/site/about/guidelines.xhtml

Major systematic reviews and meta-analyses were further scrutinized for individual trials possibly missed by the database searches.[3,5,10,11,14,29-33]

Records from PubMed and CENTRAL were combined using EndNote reference software, removing duplicate records. Titles were screened by one reviewer (H.J.) to remove apparently irrelevant publications. Abstracts and full text articles were assessed against eligibility criteria by two reviewers independently (H.J. and B.C.). Discrepancies on study inclusion were resolved by discussion or third-party involvement (M.B.). All studies fulfilling the above eligibility criteria will be reported in the systematic review, regardless of outcome reporting.

Data extraction

Data will be extracted from original publications into Excel spreadsheets by two authors independently. Extracted data will include descriptive study characteristics as well as data for analytical purposes. A preliminary list of variables to extract is presented in Table 1. Differences in extracted data will be assessed going back to original publications and, if uncertainty prevails, resolved by discussion or involvement of a third investigator.

Blood pressure data

Mean systolic and diastolic blood pressure level at baseline and during follow-up will be collected for each treatment arm in each trial. If baseline blood pressure levels are not presented for each treatment arm separately, values for the total study population will be extracted and assumed to be similar for randomized groups. For follow-up blood pressure

For peer review only

values, we will aim to include mean levels during the follow-up period. If this is not reported, we will calculate mean levels for the full follow-up manually when several values are presented, or include values at a single time point (for example last visit). Blood pressure differences between groups will be recorded as reported, or calculated manually from follow-up values when not reported separately.

Outcome data

We will extract the number of participants and events for each treatment arm in each study, whenever possible. If the number of events is not presented, we will estimate the number of events from the measure reported, e.g. calculating backwards from incidence rates, or manually counting number of events from Kaplan-Meier curves.[34] We will use the definitions described above for each outcome (see "*Outcome measures*" section).

Missing outcome data

The number of participants lost to follow-up will be recorded for cardiovascular events and mortality outcomes, for each treatment arm, separately. It is common in cardiovascular trials to follow participants for mortality through national or regional registers even if participants drop out from clinical follow-up during the intervention. Loss to follow-up will be reported descriptively, and the impact of attrition on primary outcomes will be estimated in sensitivity analyses using different assumptions for the outcome of the participants lost to follow-up allowing for some uncertainty in these assumptions.[35,36]

For peer review only - http://bmjopen.bmj.com/site/about/guidelines.xhtml

Risk of bias assessment

All eligible trials will be assessed for risk of bias using the Cochrane Collaboration's risk of bias assessment tool, second version (RoB2).[37] The RoB2 tool includes five domains relating to the randomisation process, deviations from the intended intervention, missing outcome data, measurement of the outcome, and selection of reported results. Risk of bias will be assessed in relation to assignment of intervention (intention-to-treat).

The first two domains will be assessed on study level, as the same judgement apply for all outcomes. Trials judged to be at high risk of bias in the randomisation process will be excluded from further analyses. In the second domain, PROBE trials will be considered to be at low risk of bias as long as no specific concerns are noted. The third domain will be assessed for mortality outcomes and non-mortality outcomes separately, as clinical events are usually not available for participants lost to follow-up, whereas vital status may still be available through population registers. If the number of participants with missing outcome data are of the same magnitude as the number of events, this will raise some concerns, whereas high risk of bias will generally require asymmetric missingness of such magnitude it could plausibly impact effect estimates in a clinically important way. For the fourth domain, a blinded endpoint committee will be regarded as the appropriate way of assessing outcomes. Outcome data reported as adverse events have generally been assessed in an open fashion to allow safety monitoring. This give rise to some concern, but will not be judged as high risk of bias because cardiovascular outcomes are fairly objective, and the value of blinding in such circumstances is probably limited.[38] For the fifth domain, assessing

For peer review only

potential bias from selection of reported results, deviations will be noted for specific outcomes. Assessment will be made by two investigators independently and discrepancies resolved by discussion or third-party involvement.

Data synthesis

Pairwise meta-analysis

In the presence of two or more studies comparing the same pair of interventions, we will synthesize results using random effects pairwise meta-analysis. The clinical heterogeneity (i.e. differences in study and participant characteristics) of the studies within the same treatment comparison will be evaluated prior to synthesis. We will estimate the between-study variance (τ^2) using the restricted maximum likelihood estimator. Inference on statistical heterogeneity will be based on τ^2 as well as on the I^2 statistic. Relative risks will be calculated for all outcomes in all trials according the intention-to-treat principle, or the modified intention-to-treat principle, using complete cases, where follow-up data are not available for all randomized participants. We will also perform sensitivity analyses with different assumptions about participants with incomplete data (see section sensitivity analyses).

Evaluation of the transitivity assumption

Transitivity is the fundamental assumption of NMA allowing for valid indirect inference. If transitivity is violated, results from NMA would be invalid. To evaluate transitivity, we will

For peer review only - http://bmjopen.bmj.com/site/about/guidelines.xhtml

compare the distribution of the potential effect modifiers across the available direct comparisons. Potential effect modifiers include baseline systolic blood pressure, as this has previously been associated with the effect of blood pressure lowering in general[3]; stable coronary artery disease, as this may be associated with greater benefit at low blood pressure levels[3]; diabetes mellitus, which may be associated with adverse treatment effect at low blood pressure levels[29]; and chronic kidney disease, for which the cut-off where RAAS-inhibitors are considered superior to other agents differ somewhat between different guidelines[7,8]. If the distributions of these characteristics are similar, we will infer against evidence of intransitivity. We will also assess the similarity of the node definitions (e.g. comparability of drug doses) in studies making different treatment comparisons.

Network meta-analysis

In the absence of important intransitivity, we will then perform random effects NMA. Two sets of networks will be created. Firstly, drug classes will be grouped according to the conventional classification described above, each drug class representing one node in the network. Secondly, the diuretics node will be split into one thiazide-type node and one thiazide-like node. All outcomes will be analyzed in both networks. As sensitivity analysis, we will analyze the primary efficacy and acceptability outcomes in both networks, combining the ACEi node and the ARB node into one RAAS-inhibitor node, as well as splitting the CCB node into dihydropyridine and non-dihydropyridine nodes, respectively. For each network, we will assume a common heterogeneity parameter across all comparisons.

For peer review only - http://bmjopen.bmj.com/site/about/guidelines.xhtml

For peer review only

Treatment effects will be presented for all active treatments compared to placebo, using forest plots, as well as for all comparisons within the decision set using league tables. Drug classes will be ranked, using the surface under the cumulative ranking curve (SUCRA), for each outcome separately in the two main networks, and for the primary efficacy and acceptability outcomes in the sensitivity analyses with combined RAAS-inhibitors and subdivided CCB. SUCRAs for the primary efficacy and acceptability outcomes will be presented in two-dimensional graphs.

In case several studies evaluating drug class combinations are identified, we will perform a component-level analysis assuming each drug class forming an intervention component.[39,40]

Analyses will be performed in R using the netmeta package (Ref: https://cran.r-project.org/web/packages/netmeta/netmeta.pdf).

Assessment of statistical incoherence

Incoherence will be assessed using a local and a global method: the Separating Indirect from Direct Evidence (SIDE) approach and the design-by-treatment interaction model respectively. Given that tests for incoherence often have low power, we will consider p-values smaller than 0.10 implying the presence of potential statistical incoherence.

Subgroup analyses and meta-regression

Even if important intransitivity is not obvious for the potential effect modifiers listed above (see “Evaluation of the transitivity assumption” section), potential differences in treatment effect based on the presence or absence of diabetes, coronary artery disease, and kidney

For peer review only - http://bmjopen.bmj.com/site/about/guidelines.xhtml

disease, will be explored in subgroup analyses. For this purpose, trials will be dichotomized based on if ≥50% of the included participants had each characteristic at baseline. The impact of systolic blood pressure at baseline, as well as mean age at baseline, will be assessed through meta-regression analysis.

For some trials comparing different active treatments, the blood pressure level during follow-up will differ between treatment arms. From an intention-to-treat perspective, this is not a problem, since any class that reduces blood pressure more effectively would be expected to reduce the number of events to a larger extent. However, to be able to answer the question if any drug class has blood pressure-independent effects on any outcome, such differences need to be taken into account. This will be explored in meta-regression analysis, adjusting for mean blood pressure difference between comparisons within trials.

Across-study bias

Comparison-adjusted funnel plots will be used to assess small-study effects for each outcome separately. If asymmetry is detected, we will employ network meta-regression models to test whether asymmetry is statistically significant and whether small studies tend to systematically favor specific drug classes.[41] For comparisons with ten or more studies we will also use contour-enhanced funnel plots to investigate the possibility that asymmetry is due to publication bias.

For peer review only

Confidence in network evidence

The overall confidence in the results will be assessed using the Confidence In the results from Network Meta-Analysis (CINeMA) framework.[42] This framework includes assessment of six domains; within-study bias, reporting bias, indirectness, imprecision, heterogeneity and incoherence. Taking the contribution of each trial for each comparison into account, the CINeMA tool provides a semiautomatic way of assessing the confidence in the results on comparison- as well as on network-level.

Ethics and dissemination

This review does not require ethical approval. Patients or the public were not involved in the design of this systematic review. The findings will be published in a peer-reviewed medical journal and presented at medical conferences.

For peer review only

For peer review only - http://bmjopen.bmj.com/site/about/guidelines.xhtml

Acknowledgement

Author contributions

MB conceived the idea. All authors (HJ, AC, BC and MB) contributed to the design of the study. HJ and MB drafted the manuscript. AC and BC revised the manuscript and contributed with important intellectual content. MB is the guarantor. All authors approved the final version.

Funding

This work was supported by the Heart Foundation of Northern Sweden, with no specific grant number. The funder had no role in the design of the study.

Competing interests

None

Data sharing statement

Data will be made available upon reasonable request.

For peer review only

References

1. Chow CK, Teo KK, Rangarajan S, et al. Prevalence, awareness, treatment, and control of hypertension in rural and urban communities in high-, middle-, and low-income countries. *JAMA* 2013; **310**(9): 959-68.

2. Collaborators GRF. Global, regional, and national comparative risk assessment of 84 behavioural, environmental and occupational, and metabolic risks or clusters of risks for 195 countries and territories, 1990-2017: a systematic analysis for the Global Burden of Disease Study 2017. *Lancet* 2018; **392**(10159): 1923-94.

3. Brunström M, Carlberg B. Association of Blood Pressure Lowering With Mortality and Cardiovascular Disease Across Blood Pressure Levels: A Systematic Review and Meta-analysis. *JAMA Intern Med* 2018; **178**(1): 28-36.

4. Turnbull F, Neal B, Algert C, et al. Effects of different blood-pressure-lowering regimens on major cardiovascular events: results of prospectively-designed overviews of randomised trials. *Lancet* 2003; **362**(9395): 1527-35.

5. Law MR, Morris JK, Wald NJ. Use of blood pressure lowering drugs in the prevention of cardiovascular disease: meta-analysis of 147 randomised trials in the context of expectations from prospective epidemiological studies. *British Medical Journal* 2009; **338**: 34.

6. Lindholm LH, Carlberg B, Samuelsson O. Should beta blockers remain first choice in the treatment of primary hypertension? A meta-analysis. *Lancet* 2005; **366**(9496): 1545-53.

7. Whelton PK, Carey RM, Aronow WS, et al. 2017 ACC/AHA/AAPA/ABC/ACPM/AGS/APhA/ASH/ASPC/NMA/PCNA Guideline for the Prevention,

For peer review only - http://bmjopen.bmj.com/site/about/guidelines.xhtml

Detection, Evaluation, and Management of High Blood Pressure in Adults: Executive Summary: A Report of the American College of Cardiology/American Heart Association Task Force on Clinical Practice Guidelines. *Hypertension* 2017.

8. Williams B, Mancia G, Spiering W, et al. 2018 ESC/ESH Guidelines for the management of arterial hypertension: The Task Force for the management of arterial hypertension of the European Society of Cardiology and the European Society of Hypertension: The Task Force for the management of arterial hypertension of the European Society of Cardiology and the European Society of Hypertension. *J Hypertens* 2018; **36**(10): 1953-2041.

9. Boffa RJ, Constanti M, Floyd CN, Wierzbicki AS, Committee G. Hypertension in adults: summary of updated NICE guidance. *BMJ* 2019; **367**: l5310.

10. Ettehad D, Emdin CA, Kiran A, et al. Blood pressure lowering for prevention of cardiovascular disease and death: a systematic review and meta-analysis. *Lancet* 2016; **387**(10022): 957-67.

11. Thomopoulos C, Parati G, Zanchetti A. Effects of blood pressure-lowering on outcome incidence in hypertension: 5. Head-to-head comparisons of various classes of antihypertensive drugs - overview and meta-analyses. *Journal of Hypertension* 2015; **33**(7): 1321-41.

12. Reboussin DM, Allen NB, Griswold ME, et al. Systematic Review for the 2017 ACC/AHA/AAPA/ABC/ACPM/AGS/APhA/ASH/ASPC/NMA/PCNA Guideline for the Prevention, Detection, Evaluation, and Management of High Blood Pressure in Adults: A Report of the American College of Cardiology/American Heart Association Task Force on Clinical Practice Guidelines. *Hypertension* 2017.

For peer review only

13. Mills EJ, Kanters S, Thorlund K, Chaimani A, Veroniki AA, Ioannidis JP. The effects of excluding treatments from network meta-analyses: survey. *BMJ* 2013; **347**: f5195.

14. Wei J, Galaviz KI, Kowalski AJ, et al. Comparison of Cardiovascular Events Among Users of Different Classes of Antihypertension Medications: A Systematic Review and Network Meta-analysis. *JAMA Netw Open* 2020; **3**(2): e1921618.

15. Burnier M, Bakris G, Williams B. Redefining diuretics use in hypertension: why select a thiazide-like diuretic? *J Hypertens* 2019; **37**(8): 1574-86.

16. Roush GC, Ernst ME, Kostis JB, Tandon S, Sica DA. Head-to-head comparisons of hydrochlorothiazide with indapamide and chlorthalidone: antihypertensive and metabolic effects. *Hypertension* 2015; **65**(5): 1041-6.

17. Olde Engberink RH, Frenkel WJ, van den Bogaard B, Brewster LM, Vogt L, van den Born BJ. Effects of thiazide-type and thiazide-like diuretics on cardiovascular events and mortality: systematic review and meta-analysis. *Hypertension* 2015; **65**(5): 1033-40.

18. Messerli FH, Bangalore S, Bavishi C, Rimoldi SF. Angiotensin-Converting Enzyme Inhibitors in Hypertension: To Use or Not to Use? *J Am Coll Cardiol* 2018; **71**(13): 1474-82.

19. Godfraind T. Calcium channel blockers in cardiovascular pharmacotherapy. *J Cardiovasc Pharmacol Ther* 2014; **19**(6): 501-15.

20. Higgins J, Thomas J, Chandler J, et al. Cochrane Handbook for Systematic Reviews of Interventions. 2nd ed. Chichester (UK): John Wiley & Sons; 2019.

21. Shamseer L, Moher D, Clarke M, et al. Preferred reporting items for systematic review and meta-analysis protocols (PRISMA-P) 2015: elaboration and explanation. *BMJ* 2015; **350**: g7647.

For peer review only

22. Chaimani A, Caldwell DM, Li T, Higgins JPT, Salanti G. Additional considerations are required when preparing a protocol for a systematic review with multiple interventions. *J Clin Epidemiol* 2017; **83**: 65-74.

23. Hutton B, Salanti G, Caldwell DM, et al. The PRISMA extension statement for reporting of systematic reviews incorporating network meta-analyses of health care interventions: checklist and explanations. *Ann Intern Med* 2015; **162**(11): 777-84.

24. Ponikowski P, Voors AA, Anker SD, et al. 2016 ESC Guidelines for the diagnosis and treatment of acute and chronic heart failure: The Task Force for the diagnosis and treatment of acute and chronic heart failure of the European Society of Cardiology (ESC)Developed with the special contribution of the Heart Failure Association (HFA) of the ESC. *Eur Heart J* 2016; **37**(27): 2129-200.

25. Ibanez B, James S, Agewall S, et al. 2017 ESC Guidelines for the management of acute myocardial infarction in patients presenting with ST-segment elevation: The Task Force for the management of acute myocardial infarction in patients presenting with ST-segment elevation of the European Society of Cardiology (ESC). *Eur Heart J* 2018; **39**(2): 119-77.

26. Bangalore S, Fakheri R, Toklu B, Messerli FH. Diabetes mellitus as a compelling indication for use of renin angiotensin system blockers: systematic review and meta-analysis of randomized trials. *BMJ* 2016; **352**: i438.

27. Bangalore S, Fakheri R, Wandel S, Toklu B, Wandel J, Messerli FH. Renin angiotensin system inhibitors for patients with stable coronary artery disease without heart failure: systematic review and meta-analysis of randomized trials. *Bmj-British Medical Journal* 2017; **356**.

For peer review only

For peer review only - http://bmjopen.bmj.com/site/about/guidelines.xhtml

28. Brunstrom M, Eliasson M, Nilsson PM, Carlberg B. Blood pressure treatment levels and choice of antihypertensive agent in people with diabetes mellitus: an overview of systematic reviews. *Journal of Hypertension* 2017; **35**(3): 453-62.

29. Brunström M, Carlberg B. Effect of antihypertensive treatment at different blood pressure levels in patients with diabetes mellitus: systematic review and meta-analyses. *BMJ* 2016; **352**: i717.

30. Rahimi K, Canoy D, Nazarzadeh M, et al. Investigating the stratified efficacy and safety of pharmacological blood pressure-lowering: an overall protocol for individual patient-level data meta-analyses of over 300 000 randomised participants in the new phase of the Blood Pressure Lowering Treatment Trialists' Collaboration (BPLTTC). *BMJ Open* 2019; **9**(5): e028698.

31. Thomopoulos C, Parati G, Zanchetti A. Effects of blood pressure lowering on outcome incidence in hypertension. 1. Overview, meta-analyses, and meta-regression analyses of randomized trials. *Journal of Hypertension* 2014; **32**(12): 2285-95.

32. Thomopoulos C, Parati G, Zanchetti A. Effects of blood pressure lowering on outcome incidence in hypertension: 4. Effects of various classes of antihypertensive drugs - Overview and meta-analyses. *Journal of Hypertension* 2015; **33**(2): 195-211.

33. Palmer SC, Mavridis D, Navarese E, et al. Comparative efficacy and safety of blood pressure-lowering agents in adults with diabetes and kidney disease: a network meta-analysis. *Lancet* 2015; **385**(9982): 2047-56.

34. Guyot P, Ades AE, Ouwens MJ, Welton NJ. Enhanced secondary analysis of survival data: reconstructing the data from published Kaplan-Meier survival curves. *BMC Med Res Methodol* 2012; **12**: 9.

For peer review only

35. White IR, Higgins JP, Wood AM. Allowing for uncertainty due to missing data in meta-analysis--part 1: two-stage methods. *Stat Med* 2008; **27**(5): 711-27.

36. Chaimani A, Mavridis D, Higgins JPT, Salanti G, White IR. Allowing for informative missingness in aggregate data meta-analysis with continuous or binary outcomes: Extensions to metamiss. *Stata J* 2018; **18**(3): 716-40.

37. Higgins J, Savovic J, Page M, Elbers R, Sterne J. Chapter 8: Assessing risk of bias in a randomized trial. In: Higgins J, Thomas J, Chandler J, et al., eds. Cochrane Handbook for Systematic Reviews of Interventions. 2nd ed. Chichester (UK): John Wiley & Sons; 2019: 205-28.

38. Moustgaard H, Clayton GL, Jones HE, et al. Impact of blinding on estimated treatment effects in randomised clinical trials: meta-epidemiological study. *BMJ* 2020; **368**: l6802.

39. Welton NJ, Caldwell DM, Adamopoulos E, Vedhara K. Mixed treatment comparison meta-analysis of complex interventions: psychological interventions in coronary heart disease. *Am J Epidemiol* 2009; **169**(9): 1158-65.

40. Rücker G, Petropoulou M, Schwarzer G. Network meta-analysis of multicomponent interventions. *Biom J* 2020; **62**(3): 808-21.

41. Chaimani A, Salanti G. Using network meta-analysis to evaluate the existence of small-study effects in a network of interventions. *Res Synth Methods* 2012; **3**(2): 161-76.

42. Nikolakopoulou A, Higgins JPT, Papakonstantinou T, et al. CINeMA: An approach for assessing confidence in the results of a network meta-analysis. *PLoS Med* 2020; **17**(4): e1003082.

For peer review only

Table 1 – variables for extraction

**Descriptive (study level)**	**Analytical (treatment-arm level)**
Study ID	Study ID
Publication year	Treatment (category)
No. participants randomized	Participants randomized (n)
Age (mean)	Participants followed for mortality (n)
Sex (% female)	Participants followed for CVD (n)
Baseline co-morbidities	Follow-up duration (mean)
- Cerebrovascular disease (%)	MACE (n)
- Coronary artery disease (%)	Discontinuation (n)
- Total cardiovascular disease (%)	All-cause mortality (n)
- Diabetes mellitus (%)	Cardiovascular mortality (n)
Intervention (drug and dose)	Stroke (n)
Control (drug and dose)	Myocardial infarction (n)
Funding	Heart failure (n)
Early termination	Acute Kidney Injury (n)
Reason for termination	Baseline SBP/DBP (mean)
Definition of MACE	Follow-up SBP/DBP (mean)
Definition other outcomes	SBP/DBP difference during follow-up (mean)
Comorbidity	

CVD = cardiovascular disease. MACE = major adverse cardiovascular events. SBP = systolic blood pressure. DBP = diastolic blood pressure.

For peer review only

Figure legend

Figure 1 – Network of all possible comparisons according to conventional classification. ACE = angiotensin converting enzyme inhibitors. ARB = angiotensin receptor blockers. BB = beta-blockers. CCB = calcium-channel blockers.

For peer review only

For peer review only - http://bmjopen.bmj.com/site/about/guidelines.xhtml

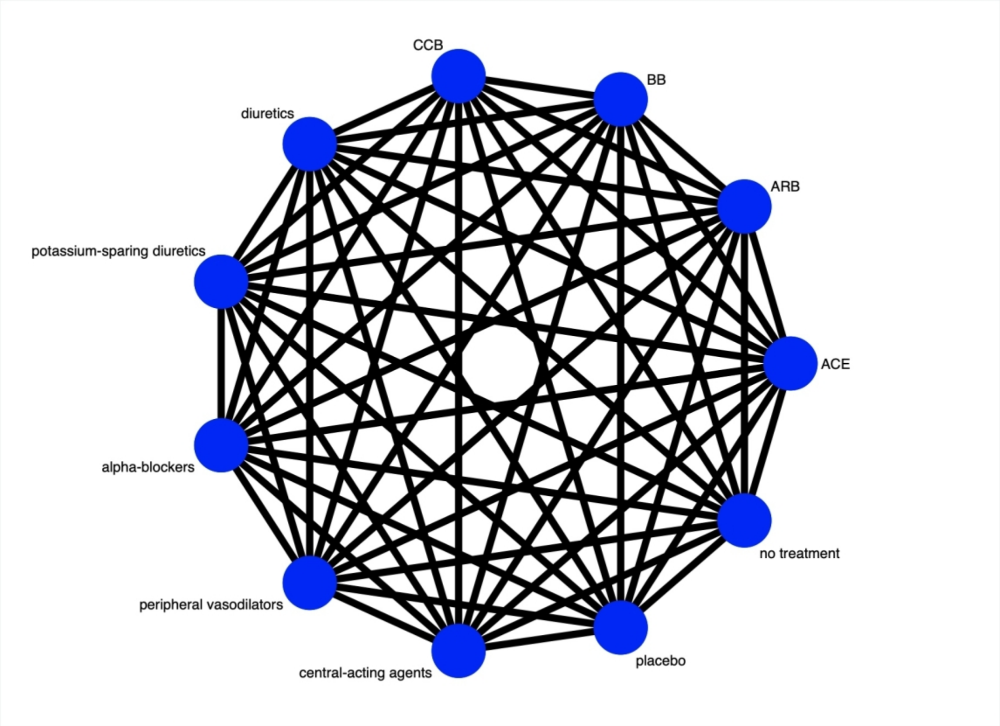

Figure 1 - Network of all possible comparisons according to conventional classification. ACE = angiotensin converting enzyme inhibitors. ARB = angiotensin receptor blockers. BB = beta-blockers. CCB = calcium-channel blockers.

320x233mm (144 x 144 DPI)

PRISMA-P 2015 Checklist

This checklist has been adapted for use with protocol submissions to *Systematic Reviews* from Table 3 in Moher D et al: Preferred reporting items for systematic review and meta-analysis protocols (PRISMA-P) 2015 statement. *Systematic Reviews* 2015 **4**:1

Section/topic	#	Checklist item	Information reported Yes	No	Line number(s)
**ADMINISTRATIVE INFORMATION**					
**Title**					
Identification	1a	Identify the report as a protocol of a systematic review	☒	☐	Page 1
Update	1b	If the protocol is for an update of a previous systematic review, identify as such	☐	☒	
**Registration**	2	If registered, provide the name of the registry (e.g., PROSPERO) and registration number in the Abstract	☒	☐	Page 2
**Authors**					
Contact	3a	Provide name, institutional affiliation, and e-mail address of all protocol authors; provide physical mailing address of corresponding author	☒	☐	Page 1
Contributions	3b	Describe contributions of protocol authors and identify the guarantor of the review	☒	☐	Page 19
**Amendments**	4	If the protocol represents an amendment of a previously completed or published protocol, identify as such and list changes; otherwise, state plan for documenting important protocol amendments	☒	☐	Page 7
**Support**					
Sources	5a	Indicate sources of financial or other support for the review	☒	☐	Page 19
Sponsor	5b	Provide name for the review funder and/or sponsor	☒	☐	Page 19
Role of sponsor/funder	5c	Describe roles of funder(s), sponsor(s), and/or institution(s), if any, in developing the protocol	☒	☐	Page 19
**INTRODUCTION**					
**Rationale**	6	Describe the rationale for the review in the context of what is already known	☒	☐	P 5-7
**Objectives**	7	Provide an explicit statement of the question(s) the review will address with reference to participants, interventions, comparators, and outcomes (PICO)	☒	☐	P 7
**METHODS**					

For peer review only

BioMed Central
The Open Access Publisher

Section/topic	#	Checklist item	Information reported Yes	Information reported No	Line number(s)
**Eligibility criteria**	8	Specify the study characteristics (e.g., PICO, study design, setting, time frame) and report characteristics (e.g., years considered, language, publication status) to be used as criteria for eligibility for the review	☒	☐	P 7-10
**Information sources**	9	Describe all intended information sources (e.g., electronic databases, contact with study authors, trial registers, or other grey literature sources) with planned dates of coverage	☒	☐	P 11
**Search strategy**	10	Present draft of search strategy to be used for at least one electronic database, including planned limits, such that it could be repeated	☒	☐	P 11
STUDY RECORDS					
Data management	11a	Describe the mechanism(s) that will be used to manage records and data throughout the review	☒	☐	P 11
Selection process	11b	State the process that will be used for selecting studies (e.g., two independent reviewers) through each phase of the review (i.e., screening, eligibility, and inclusion in meta-analysis)	☒	☐	P 12
Data collection process	11c	Describe planned method of extracting data from reports (e.g., piloting forms, done independently, in duplicate), any processes for obtaining and confirming data from investigators	☒	☐	P 12-13
**Data items**	12	List and define all variables for which data will be sought (e.g., PICO items, funding sources), any pre-planned data assumptions and simplifications	☒	☐	Table 1
**Outcomes and prioritization**	13	List and define all outcomes for which data will be sought, including prioritization of main and additional outcomes, with rationale	☒	☐	P 9-10
**Risk of bias in individual studies**	14	Describe anticipated methods for assessing risk of bias of individual studies, including whether this will be done at the outcome or study level, or both; state how this information will be used in data synthesis	☒	☐	P 13-14
DATA					
**Synthesis**	15a	Describe criteria under which study data will be quantitatively synthesized	☒	☐	P 14-16
	15b	If data are appropriate for quantitative synthesis, describe planned summary measures, methods of handling data, and methods of combining data from studies, including any planned exploration of consistency (e.g., I^2, Kendall's tau)	☒	☐	P 13 & 15-16
	15c	Describe any proposed additional analyses (e.g., sensitivity or subgroup analyses, meta-regression)	☒	☐	P 17
	15d	If quantitative synthesis is not appropriate, describe the type of summary planned	☐	☒	N/A
**Meta-bias(es)**	16	Specify any planned assessment of meta-bias(es) (e.g., publication bias across studies, selective reporting within studies)	☒	☐	P 18
**Confidence in cumulative evidence**	17	Describe how the strength of the body of evidence will be assessed (e.g., GRADE)	☒	☐	P 18

For peer review only

BioMed Central
The Open Access Publisher

BMJ Open

Comparative efficacy and acceptability of different antihypertensive drug classes for cardiovascular disease prevention – protocol for a systematic review and network meta-analysis

Journal:	*BMJ Open*
Manuscript ID	bmjopen-2020-044302.R1
Article Type:	Protocol
Date Submitted by the Author:	30-Jan-2021
Complete List of Authors:	Jussil, Heidi; Umeå Universitet Medicinska fakulteten, Department of Public Health and Clinical Medicine<br>Chaimani, Anna; Universite de Paris, Research Center of Epidemiology (CRESS-UMR1153), INSERM, INRA<br>Carlberg, Bo; Umeå Universitet Medicinska fakulteten, Department of Public Health and Clinical Medicine<br>Brunström, Mattias ; Umeå Universitet Medicinska fakulteten, Department of Public Health and Clinical Medicine
**Primary Subject Heading**:	Cardiovascular medicine
Secondary Subject Heading:	General practice / Family practice, Pharmacology and therapeutics
Keywords:	Hypertension < CARDIOLOGY, Cardiology < INTERNAL MEDICINE, Stroke medicine < INTERNAL MEDICINE, Vascular medicine < INTERNAL MEDICINE

SCHOLARONE™
Manuscripts

I, the Submitting Author has the right to grant and does grant on behalf of all authors of the Work (as defined in the below author licence), an exclusive licence and/or a non-exclusive licence for contributions from authors who are: i) UK Crown employees; ii) where BMJ has agreed a CC-BY licence shall apply, and/or iii) in accordance with the terms applicable for US Federal Government officers or employees acting as part of their official duties; on a worldwide, perpetual, irrevocable, royalty-free basis to BMJ Publishing Group Ltd ("BMJ") its licensees and where the relevant Journal is co-owned by BMJ to the co-owners of the Journal, to publish the Work in this journal and any other BMJ products and to exploit all rights, as set out in our licence.

The Submitting Author accepts and understands that any supply made under these terms is made by BMJ to the Submitting Author unless you are acting as an employee on behalf of your employer or a postgraduate student of an affiliated institution which is paying any applicable article publishing charge ("APC") for Open Access articles. Where the Submitting Author wishes to make the Work available on an Open Access basis (and intends to pay the relevant APC), the terms of reuse of such Open Access shall be governed by a Creative Commons licence – details of these licences and which Creative Commons licence will apply to this Work are set out in our licence referred to above.

Other than as permitted in any relevant BMJ Author's Self Archiving Policies, I confirm this Work has not been accepted for publication elsewhere, is not being considered for publication elsewhere and does not duplicate material already published. I confirm all authors consent to publication of this Work and authorise the granting of this licence.

For peer review only

Comparative efficacy and acceptability of different antihypertensive drug classes for cardiovascular disease prevention – protocol for a systematic review and network meta-analysis

Heidi Jussil[1], Anna Chaimani[2], Bo Carlberg[1], Mattias Brunström[1]

[1] Department of Public Health and Clinical Medicine, Umeå University, Umeå, Sweden
[2] Université de Paris, Research Center of Epidemiology and Statistics (CRESS-U1153), INSERM, INRA, F-75004 Paris, France

Correspondence:
Dr Mattias Brunström
Dept. Public Health and Clinical Medicine
Umeå University
901 87, Umeå
Sweden
mattias.brunstrom@umu.se
+46733693182

3 446 words
1 table
1 figure

For peer review only

For peer review only - http://bmjopen.bmj.com/site/about/guidelines.xhtml

Abstract

Introduction

Clinical practice guidelines differ in their recommendations on first-line antihypertensive drug classes. No adequately powered randomized controlled trial have assessed all major drug classes against each other, and previous meta-analyses have mainly relied on pairwise meta-analyses for treatment comparisons.

Methods and analysis

A systematic review and network meta-analysis will be carried out to assess the efficacy and acceptability of all major antihypertensive drug classes. PubMed and CENTRAL were searched on February 21st 2020 to identify randomized controlled trials with at least 1000 person-years of follow-up, assessing any antihypertensive agent against other agents or placebo. All trials fulfilling the inclusion criteria will be assessed for risk of bias using the second version of Cochrane's risk of bias assessment tool. The study selection process, risk of bias assessment, and data extraction is done by two authors in duplicate. Relative risks from individual trials will be combined in pairwise meta-analyses; in the absence of important intransitivity, random-effects network meta-analysis will be performed. The primary outcome for efficacy will be major adverse cardiovascular events, whereas the primary acceptability outcome will be treatment discontinuation for any reason. Additional outcomes include all-cause mortality, cardiovascular mortality, stroke, myocardial infarction, heart failure, and acute renal failure. The impact of differences within drug classes will be

For peer review only - http://bmjopen.bmj.com/site/about/guidelines.xhtml

For peer review only

explored through alternative networks, including analyzing thiazide-like and thiazide-type diuretics separately.

Ethics and dissemination

This review will only process aggregated study level data and does not require ethical approval. The findings will be published in a peer-reviewed medical journal.

Registration

The protocol was registered in PROSPERO August 21st 2020, and published September 21st 2020, with registration number CRD42020205482.

.

For peer review only

For peer review only - http://bmjopen.bmj.com/site/about/guidelines.xhtml

Strengths and limitations of this study

- This systematic review and network meta-analysis will be the first contemporary review to assess the efficacy and acceptability of all major antihypertensive drug classes, incorporating direct and indirect evidence
- Broad inclusion criteria in terms of patient characteristics ensure wide applicability of the study findings; potential differences between patient groups will be explored through subgroup- and sensitivity analyses.
- Alternative networks, with different node definitions, will be used to explore differences within drug classes, specifically differences between thiazide-like and thiazide-type diuretics.
- The main limitation of this review is the use of study-level data, with no possibility to account for individual patient characteristics in the analyses.

For peer review only

Introduction

Hypertension (blood pressure ≥ 140/90 mmHg) is estimated to affect more than one billion people globally.[1,2] Studies have shown that hypertension is the most important risk factor for death and cardiovascular disease (CVD) worldwide[2], and that lowering blood pressure in the hypertensive range reduces the risk of CVD.[2,3]

Five major drug classes have been shown to reduce the risk of CVD compared to placebo or no treatment; angiotensin-converting enzyme inhibitors (ACEi), angiotensin II receptor blockers (ARB), calcium channel blockers (CCB), diuretics and beta blockers (BB).[4,5] Although beta-blockers have generally been abandoned as first-line therapy due to less effective stroke prevention compared to other agents[6], current hypertension guidelines differ in the recommendations on what drug class to use as first-line therapy.[7-9] Whereas US guidelines recommend single therapy with ACEi, ARB, CCB or diuretics initially[7], European guidelines recommended to start treatment with a renin-angiotensin aldosterone system (RAAS) inhibitor (ACEi or ARB), in combination with a CCB or a diuretic.[8] The most recent UK recommendations, from the National Institute for Health and Care Excellence (NICE), support RAAS inhibitors in young people and people with diabetes, whereas CCB:s are considered the drug class of choice for elderly and people of African heritage.[9]

Even though many antihypertensive agents have been compared to one another or placebo separately, no adequately powered trial has compared all major drug classes head-to-head, assessing CVD outcomes. Several systematic reviews and pairwise meta-analyses aiming to assess this have been published, but all have limited their analyses to comparative trials

For peer review only

assessing two or more agents against each other, with the exclusion of placebo-controlled trials.[5,10-12] Excluding placebo-controlled trials means exclusion of potentially valuable information gained through indirect comparisons, likely affecting the overall results.[13] Further, the two most cited meta-analyses supporting the view that all major drug classes are comparable have used pairwise meta-analyses, comparing each drug class to all other drug classes.[5,10] This implicitly assumes that all drug classes used for comparison are similar, which may conceal clinically important differences. The only contemporary network meta-analysis assessing different antihypertensive agents in the general population to date, is hard to interpret due to limitations in study selection, data extraction and risk of bias assessment.[14]

All major systematic reviews have used the conventional classification of antihypertensive agents described above.[4,5,10-12] However, categorization of drug classes might play a critical role for the results of network meta-analyses. For example, the class of diuretics may be split into thiazide-type and thiazide-like diuretics. US and European guidelines equate these subclasses[7,8], whereas the UK guidelines preferentially recommend thiazide-like diuretics.[9] The latter recommendation is supported by pharmacological differences[15], as well as recent meta-analyses assessing differences in blood pressure-lowering and CVD reduction[16,17]. Another categorization of interest is that of RAAS inhibitors (ACEi and ARB). Although pharmacologically distinct, these classes act on the same physiological system, and evidence from clinical trials suggests no difference in efficacy between classes.[18] Thirdly, CCB can be subdivided into dihydropyridine and non-dihydropyridine agents, the former being highly selective for the vasculature whereas the latter also has antiarrhythmic properties.[19]

For peer review only

In summary, clinical practice guidelines differ in their recommendations on first-line antihypertensive treatment. This reflects the absence of head-to-head comparisons between major drug classes, and hence uncertainty regarding the comparative efficacy between classes. The aim and purpose of this study is to compare the efficacy and acceptability of different antihypertensive drug classes for CVD prevention through a systematic review and network meta-analysis, incorporating evidence from comparative as well as placebo-controlled trials. Further, we will explore the potential differences among different subclasses of diuretics, RAAS-inhibitors, and CCB.

Methods

We will perform a systematic review guided by the recommendations from the Cochrane Collaboration.[20] The protocol is written with guidance from the PRISMA-P statement, with additional considerations specific for network meta-analyses[21,22], and was registered in the PROSPERO database 21st of August 2020 (CRD42020205482). Any important updates to the protocol will be amended to PROSPERO, and/or described in the final report. The final report will follow the recommendations from the PRISMA-NMA statement.[23]

Eligibility criteria

Types of studies

Randomized controlled trials comparing any single antihypertensive agent, or combination of antihypertensive agents, against another agent, combination of agents, placebo, or no treatment will be included. Trials using a prospective randomized open-label blinded

For peer review only - http://bmjopen.bmj.com/site/about/guidelines.xhtml

endpoint (PROBE) design will be accepted, whereas cluster-randomized trials, crossover trials, and trials of combined or complex interventions will be excluded. Trials assessing different treatment strategies, such as more intensive versus less intensive treatment, will also be excluded, because they convey no information on drug- or drug class-specific effects. We will restrict our analyses to trials with at least 1000 person-years of follow-up to avoid small trials not primarily assessing the effect of blood pressure-lowering agents on cardiovascular events, but rather the effect of different agents on blood pressure levels. Hundreds of such trials exist; cardiovascular events are uncommon, and when present recorded as adverse events and therefore not properly adjudicated.

Types of participants

Trials will be included if participants are either primary preventive, have stable coronary artery disease, previous cerebrovascular disease, diabetes mellitus, or mild chronic kidney disease (eGFR 60-89 ml/min/1.73m^2). Because the main drug classes listed below (see "*types of intervention"* section) may be combined (except for ACEi + ARB; combination excluded), and prescribed in any order without important interactions on cardiovascular outcome level, we will include trials of treatment naïve patients and previously treated patients in the network meta-analysis. Studies including more than 50% of participants with heart failure, left ventricular dysfunction, acute myocardial infarction, macroalbuminuria, end-stage renal disease, or chronic kidney disease stage 3 or higher (eGFR < 60 ml/min/1.73m^2) at baseline will be excluded. The reason for these exclusions is that one or several of the included drug classes have specific effects in these patient groups, that possibly goes beyond blood pressure lowering.[7,8,24,25] Thus, the transitivity assumption (see "*Evaluation of the transitivity*

For peer review only - http://bmjopen.bmj.com/site/about/guidelines.xhtml

assumption" section below) would be violated if such trials were combined with trials assessing similar agents in patient populations where such effects are absent. Although some would argue for specific effects of RAAS inhibitors in people with stable coronary artery disease, as well as in people with diabetes and mild chronic kidney disease, recent systematic reviews have not confirmed such an effect on cardiovascular outcomes.[18,26-28] Based on these findings, trials in people with stable coronary artery disease, diabetes and mild chronic kidney disease will be included in our main analysis, and excluded in sensitivity analyses to assess the impact on the overall results.

Types of intervention

The main interventions of interest are antihypertensive drug classes which have previously been shown to reduce the risk of cardiovascular events; ACE, ARB, BB, CCB, and diuretics. These are the main treatment options in clinical practice for the types of participants listed above (se "*types of participants*" section), and will therefore constitute the decision set of interventions in this review. Other drug classes, such as potassium-sparing diuretics, alpha-blockers, peripheral vasodilators, and central-acting agents, as well as drug combinations including any of the above-mentioned drug classes, placebo, and no treatment, will be included in the network as supplementary interventions to maximize the amount of evidence through indirect comparisons (Figure 1).

Outcome measures

The primary outcome for efficacy will be the composite outcome, major adverse cardiovascular events (MACE). Definitions of MACE vary to some extent across trials, but

For peer review only - http://bmjopen.bmj.com/site/about/guidelines.xhtml

commonly include cardiovascular mortality, stroke, and myocardial infarction. Inclusion of heart failure, revascularisation, and peripheral artery disease differ across trials. If available, we prefer the strict definition using cardiovascular mortality, myocardial infarction, and stroke. If other definitions of MACE have been applied, they will generally be accepted but appropriateness for inclusion will be assessed on a case-by-case basis. All deviations from the preferred definition will be denoted, and the impact of MACE definition will be explored in sensitivity analyses, excluding trials not presenting results according to the preferred definition.

The primary outcome for acceptability will be permanent discontinuation of study medication. We will include discontinuations for any reason because this reflects safety as well as efficacy, and because the reporting of reasons for discontinuation differs to a large extent between trials.

Secondary outcomes are all-cause mortality, cardiovascular mortality, myocardial infarction, stroke, heart failure, and acute kidney injury. Mortality outcomes, as well as myocardial infarction and stroke, have generally accepted universal definitions, although for myocardial infarction the definition has changed slightly over the years. For these outcomes, the definitions applied in individual trials will generally be accepted. For myocardial infarction and stroke we primarily aim to include fatal and non-fatal events, combined. If only fatal or non-fatal events are reported separately, however, we will accept and include the reported numbers. We will also accept “acute coronary syndrome” as equivalent to myocardial infarction, but we will not include composite coronary events including revascularizations. For heart failure, we will accept definitions including new diagnosis of heart failure, heart

For peer review only

failure hospitalizations, and heart failure mortality. For acute kidney injury, we will apply the Kidney Disease: Improving Global Outcomes (KDIGO) definition, including at least 50 % increase in serum creatinine, where appropriate. However, we will also include discontinuation of treatment due to kidney injury, as many trials were designed before the KDIGO definition of acute kidney injury was published.

Search strategy

Preliminary searches in PubMed and CENTRAL were performed on February 21st 2020. We used search terms for blood pressure lowering treatment combined with several cardiovascular disease terms in both databases. We restricted our search to randomized controlled trials, but applied no date or language restrictions.

Full search strategy in PubMed was (("antihypertensive agents"[Pharmacological Action] OR "antihypertensive agents"[MeSH Terms] OR "antihypertensive"[All Fields] OR "blood pressure-lowering"[All Fields] OR "blood pressure lowering"[All Fields] OR "blood-pressure lowering"[All Fields]) AND ("cardiovascular"[All Fields] OR "myocardial"[All Fields] OR "stroke"[MeSH Terms] OR "stroke"[All Fields] OR "heart failure"[All Fields] OR "mortality"[All Fields]) AND (randomized controlled trial[Publication Type] OR (randomized[Title/Abstract] AND controlled[Title/Abstract] AND trial[Title/Abstract])).

Full search strategy for CENTRAL was (antihypertensive near agents OR "blood pressure-lowering" OR "blood pressure lowering" OR "blood-pressure lowering") AND ("cardiovascular" OR "myocardial" OR "stroke" OR "heart failure" OR "mortality") in All Text.

For peer review only

Major systematic reviews and meta-analyses were further scrutinized for individual trials possibly missed by the database searches.[3,5,10,11,14,29-33]

Records from PubMed and CENTRAL were combined using EndNote reference software, removing duplicate records. Titles were screened by one reviewer (H.J.) to remove apparently irrelevant publications. Abstracts and full text articles were assessed against eligibility criteria by two reviewers independently (H.J. and B.C.). Discrepancies on study inclusion were resolved by discussion or third-party involvement (M.B.). All studies fulfilling the above eligibility criteria will be reported in the systematic review, regardless of outcome reporting.

Data extraction

Data will be extracted from original publications into Excel spreadsheets by two authors independently. Extracted data will include descriptive study characteristics as well as data for analytical purposes. A preliminary list of variables to extract is presented in Table 1. Differences in extracted data will be assessed going back to original publications and, if uncertainty prevails, resolved by discussion or involvement of a third investigator.

Blood pressure data

Mean systolic and diastolic blood pressure level at baseline and during follow-up will be collected for each treatment arm in each trial. If baseline blood pressure levels are not presented for each treatment arm separately, values for the total study population will be extracted and assumed to be similar for randomized groups. For follow-up blood pressure

For peer review only

values, we will aim to include mean levels during the follow-up period. If this is not reported, we will calculate mean levels for the full follow-up manually when several values are presented, or include values at a single time point (for example last visit). Blood pressure differences between groups will be recorded as reported, or calculated manually from follow-up values when not reported separately.

Outcome data

We will extract the number of participants and events for each treatment arm in each study, whenever possible. If the number of events is not presented, we will estimate the number of events from the measure reported, e.g. calculating backwards from incidence rates, or manually counting number of events from Kaplan-Meier curves.[34] We will use the definitions described above for each outcome (see "*Outcome measures*" section).

Missing outcome data

The number of participants lost to follow-up will be recorded for cardiovascular events and mortality outcomes, for each treatment arm, separately. It is common in cardiovascular trials to follow participants for mortality through national or regional registers even if participants drop out from clinical follow-up during the intervention. Loss to follow-up will be reported descriptively, and the impact of attrition on primary outcomes will be estimated in sensitivity analyses using different assumptions for the outcome of the participants lost to follow-up allowing for some uncertainty in these assumptions.[35,36]

For peer review only

Risk of bias assessment

All eligible trials will be assessed for risk of bias using the Cochrane Collaboration's risk of bias assessment tool, second version (RoB2).[37] The RoB2 tool includes five domains relating to the randomisation process, deviations from the intended intervention, missing outcome data, measurement of the outcome, and selection of reported results. Risk of bias will be assessed in relation to assignment of intervention (intention-to-treat).

The first two domains will be assessed on study level, as the same judgement apply for all outcomes. Trials judged to be at high risk of bias in the randomisation process will be excluded from further analyses. In the second domain, PROBE trials will be considered to be at low risk of bias as long as no specific concerns are noted. The third domain will be assessed for mortality outcomes and non-mortality outcomes separately, as clinical events are usually not available for participants lost to follow-up, whereas vital status may still be available through population registers. If the number of participants with missing outcome data are of the same magnitude as the number of events, this will raise some concerns, whereas high risk of bias will generally require asymmetric missingness of such magnitude it could plausibly impact effect estimates in a clinically important way. For the fourth domain, a blinded endpoint committee will be regarded as the appropriate way of assessing outcomes. Outcome data reported as adverse events have generally been assessed in an open fashion to allow safety monitoring. This give rise to some concern, but will not be judged as high risk of bias because cardiovascular outcomes are fairly objective, and the value of blinding in such circumstances is probably limited.[38] For the fifth domain, assessing

For peer review only

potential bias from selection of reported results, deviations will be noted for specific outcomes. Assessment will be made by two investigators independently and discrepancies resolved by discussion or third-party involvement.

The risk of bias assessment will be incorporated in the overall assessment of the quality of evidence, within the CINEMA framework, as described under the subheading "Confidence in network evidence".

Data synthesis

Pairwise meta-analysis

In the presence of two or more studies comparing the same pair of interventions, we will synthesize results using random effects pairwise meta-analysis. The clinical heterogeneity (i.e. differences in study and participant characteristics) of the studies within the same treatment comparison will be evaluated prior to synthesis. We will estimate the between-study variance (τ^2) using the restricted maximum likelihood estimator. Inference on statistical heterogeneity will be based on τ^2 as well as on the I^2 statistic. Relative risks will be calculated for all outcomes in all trials according the intention-to-treat principle, or the modified intention-to-treat principle, using complete cases, where follow-up data are not available for all randomized participants. We will also perform sensitivity analyses with different assumptions about participants with incomplete data (see section sensitivity analyses).

For peer review only - http://bmjopen.bmj.com/site/about/guidelines.xhtml

Evaluation of the transitivity assumption

Transitivity is the fundamental assumption of NMA allowing for valid indirect inference. If transitivity is violated, results from NMA would be invalid. To evaluate transitivity, we will compare the distribution of the potential effect modifiers across the available direct comparisons. Potential effect modifiers include baseline systolic blood pressure, as this has previously been associated with the effect of blood pressure lowering in general[3]; stable coronary artery disease, as this may be associated with greater benefit at low blood pressure levels[3]; diabetes mellitus, which may be associated with adverse treatment effect at low blood pressure levels[29]; and chronic kidney disease, for which the cut-off where RAAS-inhibitors are considered superior to other agents differ somewhat between different guidelines[7,8]. If the distributions of these characteristics are similar, we will infer against evidence of intransitivity. We will also assess the similarity of the node definitions (e.g. comparability of drug doses) in studies making different treatment comparisons.

Network meta-analysis

In the absence of important intransitivity, we will then perform random effects NMA. Two sets of networks will be created. Firstly, drug classes will be grouped according to the conventional classification described above, each drug class representing one node in the network. Secondly, the diuretics node will be split into one thiazide-type node and one thiazide-like node. All outcomes will be analyzed in both networks. As sensitivity analyses, we will analyze the primary efficacy and acceptability outcomes in both networks, combining the ACEi node and the ARB node into one RAAS-inhibitor node, as well as splitting the CCB

For peer review only

node into dihydropyridine and non-dihydropyridine nodes, respectively. For each network, we will assume a common heterogeneity parameter across all comparisons.

Treatment effects will be presented for all active treatments compared to placebo, using forest plots, as well as for all comparisons within the decision set using league tables. Drug classes will be ranked, using the surface under the cumulative ranking curve (SUCRA), for each outcome separately in the two main networks, and for the primary efficacy and acceptability outcomes in the sensitivity analyses with combined RAAS-inhibitors and subdivided CCB. SUCRAs for the primary efficacy and acceptability outcomes will be presented in two-dimensional graphs.

In case several studies evaluating drug class combinations are identified, we will perform a component-level analysis assuming each drug class forming an intervention component.[39,40]

Analyses will be performed in R using the netmeta package (Ref: https://cran.r-project.org/web/packages/netmeta/netmeta.pdf).

Assessment of statistical incoherence

Incoherence will be assessed using a local and a global method: the Separating Indirect from Direct Evidence (SIDE) approach and the design-by-treatment interaction model respectively. Given that tests for incoherence often have low power, we will consider p-values smaller than 0.10 implying the presence of potential statistical incoherence.

For peer review only - http://bmjopen.bmj.com/site/about/guidelines.xhtml

Subgroup analyses and meta-regression

Even if important intransitivity is not obvious for the potential effect modifiers listed above (see "Evaluation of the transitivity assumption" section), potential differences in treatment effect based on the presence or absence of diabetes, coronary artery disease, and kidney disease, will be explored in subgroup or meta-regression analyses. Although meta-regression is more powerful in detecting associations between trial characterises and treatment effect compared to subgroup analyses, our experience is that many trials do not report the exact percentage of participants with certain diseases. It may still be clear from the methods, or from other numbers presented, that it is well below certain thresholds. Thus, meta-regression will be used if permitted by the available data, otherwise trials will be dichotomized based on if ≥50% of the included participants had each characteristic at baseline. The impact of systolic blood pressure at baseline, as well as mean age at baseline, will be assessed through meta-regression analysis.

For some trials comparing different active treatments, the blood pressure level during follow-up will differ between treatment arms. From an intention-to-treat perspective, this is not a problem, since any class that reduces blood pressure more effectively would be expected to reduce the number of events to a larger extent. However, to be able to answer the question if any drug class has blood pressure-independent effects on any outcome, such differences need to be taken into account. This will be explored in meta-regression analysis, adjusting for mean blood pressure difference between comparisons within trials.

For peer review only - http://bmjopen.bmj.com/site/about/guidelines.xhtml

Across-study bias

Comparison-adjusted funnel plots will be used to assess small-study effects for each outcome separately. If asymmetry is detected, we will employ network meta-regression models to test whether asymmetry is statistically significant and whether small studies tend to systematically favor specific drug classes.[41] For comparisons with ten or more studies we will also use contour-enhanced funnel plots to investigate the possibility that asymmetry is due to publication bias.

Confidence in network evidence

The overall confidence in the results will be assessed using the Confidence In the results from Network Meta-Analysis (CINeMA) framework.[42] This framework includes assessment of six domains; within-study bias, reporting bias, indirectness, imprecision, heterogeneity and incoherence. Taking the contribution of each trial for each comparison into account, the CINeMA tool provides a semiautomatic way of assessing the confidence in the results on comparison- as well as on network-level.

Ethics and dissemination

This review does not require ethical approval. The findings will be published in a peer-reviewed medical journal and presented at medical conferences.

For peer review only - http://bmjopen.bmj.com/site/about/guidelines.xhtml

Patient and Public Involvement

No patient of public involvement.

For peer review only

For peer review only - http://bmjopen.bmj.com/site/about/guidelines.xhtml

Acknowledgement

Author contributions

MB conceived the idea. All authors (HJ, AC, BC and MB) contributed to the design of the study. HJ and MB drafted the manuscript. AC and BC revised the manuscript and contributed with important intellectual content. MB is the guarantor. All authors approved the final version.

Funding

This work was supported by the Heart Foundation of Northern Sweden, with no specific grant number. The funder had no role in the design of the study.

Competing interests

None

Data sharing statement

Data will be made available upon reasonable request.

For peer review only

References

1. Chow CK, Teo KK, Rangarajan S, et al. Prevalence, awareness, treatment, and control of hypertension in rural and urban communities in high-, middle-, and low-income countries. *JAMA* 2013; **310**(9): 959-68.

2. Collaborators GRF. Global, regional, and national comparative risk assessment of 84 behavioural, environmental and occupational, and metabolic risks or clusters of risks for 195 countries and territories, 1990-2017: a systematic analysis for the Global Burden of Disease Study 2017. *Lancet* 2018; **392**(10159): 1923-94.

3. Brunström M, Carlberg B. Association of Blood Pressure Lowering With Mortality and Cardiovascular Disease Across Blood Pressure Levels: A Systematic Review and Meta-analysis. *JAMA Intern Med* 2018; **178**(1): 28-36.

4. Turnbull F, Neal B, Algert C, et al. Effects of different blood-pressure-lowering regimens on major cardiovascular events: results of prospectively-designed overviews of randomised trials. *Lancet* 2003; **362**(9395): 1527-35.

5. Law MR, Morris JK, Wald NJ. Use of blood pressure lowering drugs in the prevention of cardiovascular disease: meta-analysis of 147 randomised trials in the context of expectations from prospective epidemiological studies. *British Medical Journal* 2009; **338**: 34.

6. Lindholm LH, Carlberg B, Samuelsson O. Should beta blockers remain first choice in the treatment of primary hypertension? A meta-analysis. *Lancet* 2005; **366**(9496): 1545-53.

7. Whelton PK, Carey RM, Aronow WS, et al. 2017 ACC/AHA/AAPA/ABC/ACPM/AGS/APhA/ASH/ASPC/NMA/PCNA Guideline for the Prevention,

For peer review only - http://bmjopen.bmj.com/site/about/guidelines.xhtml

Detection, Evaluation, and Management of High Blood Pressure in Adults: Executive Summary: A Report of the American College of Cardiology/American Heart Association Task Force on Clinical Practice Guidelines. *Hypertension* 2017.

8. Williams B, Mancia G, Spiering W, et al. 2018 ESC/ESH Guidelines for the management of arterial hypertension: The Task Force for the management of arterial hypertension of the European Society of Cardiology and the European Society of Hypertension: The Task Force for the management of arterial hypertension of the European Society of Cardiology and the European Society of Hypertension. *J Hypertens* 2018; **36**(10): 1953-2041.

9. Boffa RJ, Constanti M, Floyd CN, Wierzbicki AS, Committee G. Hypertension in adults: summary of updated NICE guidance. *BMJ* 2019; **367**: l5310.

10. Ettehad D, Emdin CA, Kiran A, et al. Blood pressure lowering for prevention of cardiovascular disease and death: a systematic review and meta-analysis. *Lancet* 2016; **387**(10022): 957-67.

11. Thomopoulos C, Parati G, Zanchetti A. Effects of blood pressure-lowering on outcome incidence in hypertension: 5. Head-to-head comparisons of various classes of antihypertensive drugs - overview and meta-analyses. *Journal of Hypertension* 2015; **33**(7): 1321-41.

12. Reboussin DM, Allen NB, Griswold ME, et al. Systematic Review for the 2017 ACC/AHA/AAPA/ABC/ACPM/AGS/APhA/ASH/ASPC/NMA/PCNA Guideline for the Prevention, Detection, Evaluation, and Management of High Blood Pressure in Adults: A Report of the American College of Cardiology/American Heart Association Task Force on Clinical Practice Guidelines. *Hypertension* 2017.

For peer review only - http://bmjopen.bmj.com/site/about/guidelines.xhtml

13. Mills EJ, Kanters S, Thorlund K, Chaimani A, Veroniki AA, Ioannidis JP. The effects of excluding treatments from network meta-analyses: survey. *BMJ* 2013; **347**: f5195.

14. Wei J, Galaviz KI, Kowalski AJ, et al. Comparison of Cardiovascular Events Among Users of Different Classes of Antihypertension Medications: A Systematic Review and Network Meta-analysis. *JAMA Netw Open* 2020; **3**(2): e1921618.

15. Burnier M, Bakris G, Williams B. Redefining diuretics use in hypertension: why select a thiazide-like diuretic? *J Hypertens* 2019; **37**(8): 1574-86.

16. Roush GC, Ernst ME, Kostis JB, Tandon S, Sica DA. Head-to-head comparisons of hydrochlorothiazide with indapamide and chlorthalidone: antihypertensive and metabolic effects. *Hypertension* 2015; **65**(5): 1041-6.

17. Olde Engberink RH, Frenkel WJ, van den Bogaard B, Brewster LM, Vogt L, van den Born BJ. Effects of thiazide-type and thiazide-like diuretics on cardiovascular events and mortality: systematic review and meta-analysis. *Hypertension* 2015; **65**(5): 1033-40.

18. Messerli FH, Bangalore S, Bavishi C, Rimoldi SF. Angiotensin-Converting Enzyme Inhibitors in Hypertension: To Use or Not to Use? *J Am Coll Cardiol* 2018; **71**(13): 1474-82.

19. Godfraind T. Calcium channel blockers in cardiovascular pharmacotherapy. *J Cardiovasc Pharmacol Ther* 2014; **19**(6): 501-15.

20. Higgins J, Thomas J, Chandler J, et al. Cochrane Handbook for Systematic Reviews of Interventions. 2nd ed. Chichester (UK): John Wiley & Sons; 2019.

21. Shamseer L, Moher D, Clarke M, et al. Preferred reporting items for systematic review and meta-analysis protocols (PRISMA-P) 2015: elaboration and explanation. *BMJ* 2015; **350**: g7647.

For peer review only

22. Chaimani A, Caldwell DM, Li T, Higgins JPT, Salanti G. Additional considerations are required when preparing a protocol for a systematic review with multiple interventions. *J Clin Epidemiol* 2017; **83**: 65-74.

23. Hutton B, Salanti G, Caldwell DM, et al. The PRISMA extension statement for reporting of systematic reviews incorporating network meta-analyses of health care interventions: checklist and explanations. *Ann Intern Med* 2015; **162**(11): 777-84.

24. Ponikowski P, Voors AA, Anker SD, et al. 2016 ESC Guidelines for the diagnosis and treatment of acute and chronic heart failure: The Task Force for the diagnosis and treatment of acute and chronic heart failure of the European Society of Cardiology (ESC)Developed with the special contribution of the Heart Failure Association (HFA) of the ESC. *Eur Heart J* 2016; **37**(27): 2129-200.

25. Ibanez B, James S, Agewall S, et al. 2017 ESC Guidelines for the management of acute myocardial infarction in patients presenting with ST-segment elevation: The Task Force for the management of acute myocardial infarction in patients presenting with ST-segment elevation of the European Society of Cardiology (ESC). *Eur Heart J* 2018; **39**(2): 119-77.

26. Bangalore S, Fakheri R, Toklu B, Messerli FH. Diabetes mellitus as a compelling indication for use of renin angiotensin system blockers: systematic review and meta-analysis of randomized trials. *BMJ* 2016; **352**: i438.

27. Bangalore S, Fakheri R, Wandel S, Toklu B, Wandel J, Messerli FH. Renin angiotensin system inhibitors for patients with stable coronary artery disease without heart failure: systematic review and meta-analysis of randomized trials. *Bmj-British Medical Journal* 2017; **356**.

For peer review only - http://bmjopen.bmj.com/site/about/guidelines.xhtml

28. Brunstrom M, Eliasson M, Nilsson PM, Carlberg B. Blood pressure treatment levels and choice of antihypertensive agent in people with diabetes mellitus: an overview of systematic reviews. *Journal of Hypertension* 2017; **35**(3): 453-62.

29. Brunström M, Carlberg B. Effect of antihypertensive treatment at different blood pressure levels in patients with diabetes mellitus: systematic review and meta-analyses. *BMJ* 2016; **352**: i717.

30. Rahimi K, Canoy D, Nazarzadeh M, et al. Investigating the stratified efficacy and safety of pharmacological blood pressure-lowering: an overall protocol for individual patient-level data meta-analyses of over 300 000 randomised participants in the new phase of the Blood Pressure Lowering Treatment Trialists' Collaboration (BPLTTC). *BMJ Open* 2019; **9**(5): e028698.

31. Thomopoulos C, Parati G, Zanchetti A. Effects of blood pressure lowering on outcome incidence in hypertension. 1. Overview, meta-analyses, and meta-regression analyses of randomized trials. *Journal of Hypertension* 2014; **32**(12): 2285-95.

32. Thomopoulos C, Parati G, Zanchetti A. Effects of blood pressure lowering on outcome incidence in hypertension: 4. Effects of various classes of antihypertensive drugs - Overview and meta-analyses. *Journal of Hypertension* 2015; **33**(2): 195-211.

33. Palmer SC, Mavridis D, Navarese E, et al. Comparative efficacy and safety of blood pressure-lowering agents in adults with diabetes and kidney disease: a network meta-analysis. *Lancet* 2015; **385**(9982): 2047-56.

34. Guyot P, Ades AE, Ouwens MJ, Welton NJ. Enhanced secondary analysis of survival data: reconstructing the data from published Kaplan-Meier survival curves. *BMC Med Res Methodol* 2012; **12**: 9.

For peer review only

For peer review only - http://bmjopen.bmj.com/site/about/guidelines.xhtml

35. White IR, Higgins JP, Wood AM. Allowing for uncertainty due to missing data in meta-analysis--part 1: two-stage methods. *Stat Med* 2008; **27**(5): 711-27.

36. Chaimani A, Mavridis D, Higgins JPT, Salanti G, White IR. Allowing for informative missingness in aggregate data meta-analysis with continuous or binary outcomes: Extensions to metamiss. *Stata J* 2018; **18**(3): 716-40.

37. Higgins J, Savovic J, Page M, Elbers R, Sterne J. Chapter 8: Assessing risk of bias in a randomized trial. In: Higgins J, Thomas J, Chandler J, et al., eds. Cochrane Handbook for Systematic Reviews of Interventions. 2nd ed. Chichester (UK): John Wiley & Sons; 2019: 205-28.

38. Moustgaard H, Clayton GL, Jones HE, et al. Impact of blinding on estimated treatment effects in randomised clinical trials: meta-epidemiological study. *BMJ* 2020; **368**: l6802.

39. Welton NJ, Caldwell DM, Adamopoulos E, Vedhara K. Mixed treatment comparison meta-analysis of complex interventions: psychological interventions in coronary heart disease. *Am J Epidemiol* 2009; **169**(9): 1158-65.

40. Rücker G, Petropoulou M, Schwarzer G. Network meta-analysis of multicomponent interventions. *Biom J* 2020; **62**(3): 808-21.

41. Chaimani A, Salanti G. Using network meta-analysis to evaluate the existence of small-study effects in a network of interventions. *Res Synth Methods* 2012; **3**(2): 161-76.

42. Nikolakopoulou A, Higgins JPT, Papakonstantinou T, et al. CINeMA: An approach for assessing confidence in the results of a network meta-analysis. *PLoS Med* 2020; **17**(4): e1003082.

For peer review only

For peer review only - http://bmjopen.bmj.com/site/about/guidelines.xhtml

Table 1 – variables for extraction

**Descriptive (study level)**	**Analytical (treatment-arm level)**
Study ID	Study ID
Publication year	Treatment (category)
No. participants randomized	Participants randomized (n)
Age (mean)	Participants followed for mortality (n)
Sex (% female)	Participants followed for CVD (n)
Baseline co-morbidities	Follow-up duration (mean)
- Cerebrovascular disease (%)	MACE (n)
- Coronary artery disease (%)	Discontinuation (n)
- Total cardiovascular disease (%)	All-cause mortality (n)
- Diabetes mellitus (%)	Cardiovascular mortality (n)
Intervention (drug and dose)	Stroke (n)
Control (drug and dose)	Myocardial infarction (n)
Funding	Heart failure (n)
Early termination	Acute Kidney Injury (n)
Reason for termination	Baseline SBP/DBP (mean)
Definition of MACE	Follow-up SBP/DBP (mean)
Definition other outcomes	SBP/DBP difference during follow-up (mean)
Comorbidity	

CVD = cardiovascular disease. MACE = major adverse cardiovascular events. SBP = systolic blood pressure. DBP = diastolic blood pressure.

For peer review only

For peer review only - http://bmjopen.bmj.com/site/about/guidelines.xhtml

Figure legend

Figure 1 – Network of all possible comparisons according to conventional classification. ACE = angiotensin converting enzyme inhibitors. ARB = angiotensin receptor blockers. BB = beta-blockers. CCB = calcium-channel blockers.

For peer review only

For peer review only - http://bmjopen.bmj.com/site/about/guidelines.xhtml

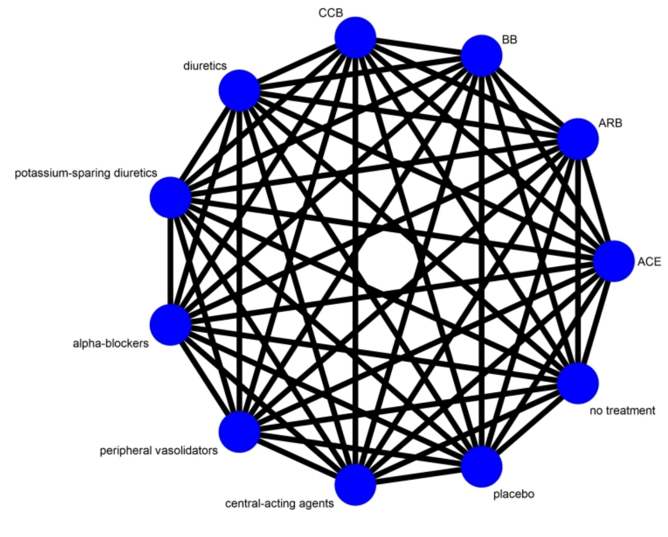

Figure 1 - Network of all possible comparisons according to conventional classification. ACE = angiotensin converting enzyme inhibitors. ARB = angiotensin receptor blockers. BB = beta-blockers. CCB = calcium-channel blockers.

338x190mm (300 x 300 DPI)

PRISMA-P 2015 Checklist

This checklist has been adapted for use with protocol submissions to *Systematic Reviews* from Table 3 in Moher D et al: Preferred reporting items for systematic review and meta-analysis protocols (PRISMA-P) 2015 statement. *Systematic Reviews* 2015 **4**:1

Section/topic	#	Checklist item	Information reported		Line number(s)
			Yes	No	
**ADMINISTRATIVE INFORMATION**					
**Title**					
Identification	1a	Identify the report as a protocol of a systematic review	☒	☐	Page 1
Update	1b	If the protocol is for an update of a previous systematic review, identify as such	☐	☒	
**Registration**	2	If registered, provide the name of the registry (e.g., PROSPERO) and registration number in the Abstract	☒	☐	Page 2
**Authors**					
Contact	3a	Provide name, institutional affiliation, and e-mail address of all protocol authors; provide physical mailing address of corresponding author	☒	☐	Page 1
Contributions	3b	Describe contributions of protocol authors and identify the guarantor of the review	☒	☐	Page 19
**Amendments**	4	If the protocol represents an amendment of a previously completed or published protocol, identify as such and list changes; otherwise, state plan for documenting important protocol amendments	☒	☐	Page 7
**Support**					
Sources	5a	Indicate sources of financial or other support for the review	☒	☐	Page 19
Sponsor	5b	Provide name for the review funder and/or sponsor	☒	☐	Page 19
Role of sponsor/funder	5c	Describe roles of funder(s), sponsor(s), and/or institution(s), if any, in developing the protocol	☒	☐	Page 19
**INTRODUCTION**					
**Rationale**	6	Describe the rationale for the review in the context of what is already known	☒	☐	P 5-7
**Objectives**	7	Provide an explicit statement of the question(s) the review will address with reference to participants, interventions, comparators, and outcomes (PICO)	☒	☐	P 7
**METHODS**					

For peer review only

BioMed Central The Open Access Publisher

Section/topic	#	Checklist item	Information reported Yes	Information reported No	Line number(s)
**Eligibility criteria**	8	Specify the study characteristics (e.g., PICO, study design, setting, time frame) and report characteristics (e.g., years considered, language, publication status) to be used as criteria for eligibility for the review	☒	☐	P 7-10
**Information sources**	9	Describe all intended information sources (e.g., electronic databases, contact with study authors, trial registers, or other grey literature sources) with planned dates of coverage	☒	☐	P 11
**Search strategy**	10	Present draft of search strategy to be used for at least one electronic database, including planned limits, such that it could be repeated	☒	☐	P 11
STUDY RECORDS					
Data management	11a	Describe the mechanism(s) that will be used to manage records and data throughout the review	☒	☐	P 11
Selection process	11b	State the process that will be used for selecting studies (e.g., two independent reviewers) through each phase of the review (i.e., screening, eligibility, and inclusion in meta-analysis)	☒	☐	P 12
Data collection process	11c	Describe planned method of extracting data from reports (e.g., piloting forms, done independently, in duplicate), any processes for obtaining and confirming data from investigators	☒	☐	P 12-13
**Data items**	12	List and define all variables for which data will be sought (e.g., PICO items, funding sources), any pre-planned data assumptions and simplifications	☒	☐	Table 1
**Outcomes and prioritization**	13	List and define all outcomes for which data will be sought, including prioritization of main and additional outcomes, with rationale	☒	☐	P 9-10
**Risk of bias in individual studies**	14	Describe anticipated methods for assessing risk of bias of individual studies, including whether this will be done at the outcome or study level, or both; state how this information will be used in data synthesis	☒	☐	P 13-14
DATA					
**Synthesis**	15a	Describe criteria under which study data will be quantitatively synthesized	☒	☐	P 14-16
	15b	If data are appropriate for quantitative synthesis, describe planned summary measures, methods of handling data, and methods of combining data from studies, including any planned exploration of consistency (e.g., I^2, Kendall's tau)	☒	☐	P 13 & 15-16
	15c	Describe any proposed additional analyses (e.g., sensitivity or subgroup analyses, meta-regression)	☒	☐	P 17
	15d	If quantitative synthesis is not appropriate, describe the type of summary planned	☐	☒	N/A
**Meta-bias(es)**	16	Specify any planned assessment of meta-bias(es) (e.g., publication bias across studies, selective reporting within studies)	☒	☐	P 18
**Confidence in cumulative evidence**	17	Describe how the strength of the body of evidence will be assessed (e.g., GRADE)	☒	☐	P 18

BioMed Central The Open Access Publisher
